# Can a management pathway for chronic cough in children improve clinical outcomes: protocol for a multicentre evaluation

**DOI:** 10.1186/1745-6215-11-103

**Published:** 2010-11-06

**Authors:** AB Chang, CF Robertson, PP van Asperen, NJ Glasgow, IB Masters, CM Mellis, LI Landau, L Teoh, PS Morris

**Affiliations:** 1 Child Health Division, Menzies School of Health Research, Charles Darwin University, Darwin, Northern Territory, Australia; 2 Queensland Children's Respiratory Centre and Queensland Children's Medical Research Institute, Royal Children's Hospital, Brisbane, Qld, Australia; 3 Department of Respiratory Medicine, Royal Children's Hospital, Murdoch Children's Research Institute, University of Melbourne, Melbourne, Victoria, Australia; 4 Department of Respiratory Medicine, The Children's Hospital at Westmead, University of Sydney, NSW, Australia; 5 Medicine School, Australian National University, Canberra, Australian Capital Territory, Australia; 6 Central Clinical School, University of Sydney, NSW, Australia; 7 Postgraduate Medical Council of Western Australia, Health Department of Western Australia, Perth, Australia; 8 The Canberra Hospital, Australian Capital Territory, Australia

## Abstract

**Background:**

Chronic cough is common and is associated with significant economic and human costs. While cough can be a problematic symptom without serious consequences, it could also reflect a serious underlying illness. Evidence shows that the management of chronic cough in children needs to be improved. Our study tests the hypothesis that the management of chronic cough in children with an evidence-based management pathway is feasible and reliable, and improves clinical outcomes.

**Methods/Design:**

We are conducting a multicentre randomised controlled trial based in respiratory clinics in 5 major Australian cities. Children (n = 250) fulfilling inclusion criteria (new patients with chronic cough) are randomised (allocation concealed) to the standardised clinical management pathway (specialist starts clinical pathway within 2 weeks) or usual care (existing care until review by specialist at 6 weeks). Cough diary, cough-specific quality of life (QOL) and generic QOL are collected at baseline and at 6, 10, 14, 26, and 52 weeks. Children are followed-up for 6 months after diagnosis and cough resolution (with at least monthly contact from study nurses). A random sample from each site will be independently examined to determine adherence to the pathway. Primary outcomes are group differences in QOL and proportion of children that are cough free at week 6.

**Discussion:**

The clinical management pathway is based on data from Cochrane Reviews combined with collective clinical experience (250 doctor years). This study will provide additional evidence on the optimal management of chronic cough in children.

**Trial registration:**

ACTRN12607000526471

## Background

Cough is the most common symptom presenting to primary care in Australia and internationally [1,2]. It is one of the most common reasons for referral to respiratory physicians. The burden of the symptom is considerable- both in terms of personal cost (e.g. impaired quality of life and multiple doctor consultations)[3] and at a societal level (through absences from school and work[4,5] and substantial medication expenses) [6]. Our previous studies have shown that >80% of parents of children with chronic cough have sought ≥5 medical consultations prior to referral [3]. Furthermore, ignoring cough (which may be the sole presenting symptom of an underlying respiratory illness) could lead to progression of a serious illness (such as bronchiectasis or retained foreign body in the airways) [7,8]. Paradoxically, cough is poorly researched. Patients are often inappropriately investigated and managed [9]. The need to improve the management of chronic cough in children is reflected in international[10-12] and Australian data [4,9], and our own collective experience.

The use of guidelines, recommendations, and clinical pathways can improve the quality of care [13,14]. Successful development of clinical guidelines requires many strategies. Initially, endorsement from experts and high quality of the evidence are important [15]. However, the use of guidelines is not universally popular in medical circles. "Numerous clinicians consider the compliance with tightened standards of care an impediment to their therapeutic freedom by imposing 'cookbook medicine' as a dogma" [14]. Also, provision of guidelines alone are not necessarily sufficient to change practice. However, the case for changing practice to be consistent with guidelines is much greater when the guidelines have been shown to improve patient outcomes. While multiple cough pathways exist, none has been subjected to a randomised controlled study [16]. Currently, the potential benefit of this type of management remains unclear. In our study, we will evaluate the feasibility and effectiveness of an evidenced-based clinical management pathway.

### Aims of the study

Our primary question in conducting the study is: Among Indigenous and non-Indigenous children with chronic cough, does management according to a standardised clinical management pathway (compared to usual treatment) improve clinical outcomes by 6 weeks? Our secondary aims are to:

1. Assess the reliability and validity of a standardised clinical management pathway for chronic cough in children; and

2. Compare the outcomes of chronic cough in Indigenous children with non-Indigenous children.

Our hypothesis is: The management of chronic cough in children in accordance with a evidence-based management pathway is feasible, reliable and improves clinical outcomes.

## Methods/Design

### Study design

We are conducting a pragmatic multi-centre randomised controlled trial (RCT) in urban respiratory specialist clinics in 5 major Australian centres: Brisbane, Melbourne, Sydney, Canberra and Darwin.

### Eligibility

Inclusion criteria: Children aged <18 years with chronic cough (>4 weeks duration)referred to a respiratory specialist for the first time. Exclusion criteria: Children with a known chronic respiratory illness previously diagnosed by a respiratory physician (eg cystic fibrosis).

### Recruitment

At each site, all referrals are screened by the site-specific study nurse. Parents are informed of the usual waiting times (6-8 weeks) and invited to participate. After informed consent, participants are randomised to either the active or control arms.

### Intervention and follow up

**Figure 1:**
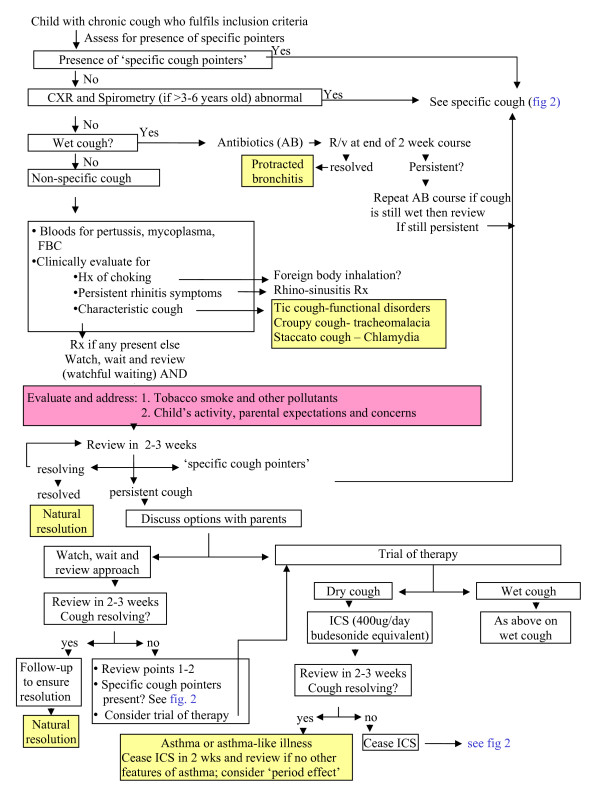
**Pathway at entry point and non-specific cough**. Entry point of cough pathway and management for non-specific cough.

**Figure 2:**
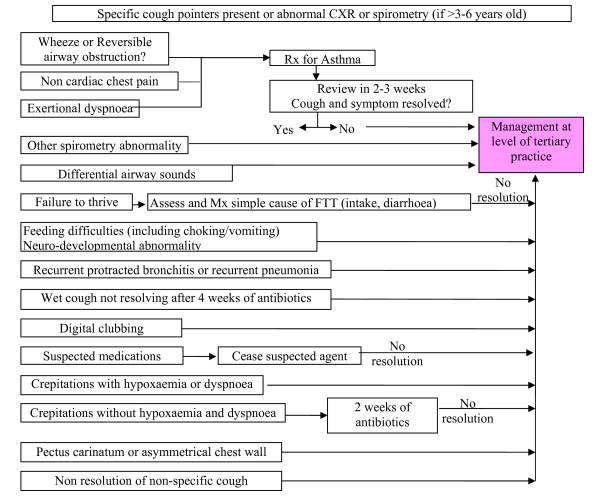
**Specific cough**. Pathway when specific cough pointers or abnormal CXR or spirometry (if aged >3-6 yrs) are present.

Children in the active arm enter the clinical pathway within 2 weeks of consent. They are managed according to the clinical pathway for at least 4 weeks before reassessment at 6 weeks (primary outcome). Children in the control arm enter the pathway at the usual waiting time (6 to 8 weeks). Since all of the participating clinics usually have a waiting time of at least 6 weeks, the control arm essentially represents usual practice. The enrolled child is managed according to the clinical pathway (figures 1 and 2). Each child is followed clinically until a primary diagnosis and cough resolution are achieved (max 12 months). Following cough resolution, children are followed-up (with monthly contacts) by the research nurse for a further 6 months. This follow-up is essential to document any recurrence of chronic cough and any additional respiratory diagnoses. To assess adherence to the pathway, a random sample of children's records (n = 10 to 20 per major participating centre: Brisbane, Sydney, Melbourne) will be examined.

### Randomisation, allocation and blinding

The randomisation sequence was computer generated and used permutated blocks (4 or 6 participants per block). Children are randomised within two age strata (≤6years and >6years) and 5 site strata (Brisbane, Melbourne, Sydney, Canberra and Darwin). In Darwin, children are also be stratified by Aboriginality (as Aboriginal children in the Northern Territory are at an increased risk of chronic respiratory disease) [17]. The allocation sequence is concealed from all the investigators, participants and caregivers as done previously [18]. All primary outcome data are collected by telephone or by interview. The participants are informed (at each contact) that the person they speak to on the telephone should only be provided with direct answers to their questions. They are also advised that they can ring the clinical research staff directly if they need any additional information. The research nurse responsible for the primary outcome data collection received training on how to ensure the validity of clinical measurements. Through these means, we are confident that the person collecting the primary outcome data remain unaware of the allocation status of the participating child (outcome assessor blinded).

#### Data collection

All data are collected on standardized forms. Outcomes (PC-QOL and PedsQL) are collected at times weeks 0 (baseline), 6, 10, 14, 26, 52 and at study endpoint. Additionally, daily cough diary scores are collected till cough resolves. Demographic data (smoking, anthropometry, etc) and medical details are collected at baseline.

#### End point

Participation is complete when the primary diagnosis is established and cough resolved, in the presence of exit criteria, or at 12 months from time of enrolment (whatever is the earliest occurrence). Exit criteria are defined as child hospitalised for condition related to cough before primary diagnosis is made (eg. child has hypoxaemia related to interstitial lung disease), or child sought treatment elsewhere for cough. Children satisfying the exit criteria are considered 'clinical failures'.

### Definitions used for clinical management pathway

• Asthma: Recurrent (>2) episodes of wheeze and/or dyspnoea that responds (within mins) to inhaled beta_2 _agonist, or bronchodilator resposiveness documented on spirometry (≥12% change in FEV_1 _%predicted after 400 ug salbutamol).

• Cough resolution: Improvement of ≥75% or total resolution according to cough diary data for ≥3 consecutive days [19,20]. When cough diary data are unavailable, resolution is defined as total cessation of verbal reporting of cough.

• CXR abnormality: Any abnormality (other than peribronchial thickening) as interpreted by specialist (respiratory or radiology).

• Primary diagnosis of aetiology of cough: Diagnosis confirmed by subsequent specific treatment which resulted in cough resolution within the expected time frame of <3weeks[20,21] (see above for time frame chosen). Diagnostic criteria are defined a-priori as published [22].

• Protracted bronchitis: (a) Presence of isolated chronic moist cough; (b) Resolution of cough with appropriate antibiotics; and (c) absence of an alternative cause for specific cough [20].

• Recurrent protracted bronchitis: ≥3 episodes per year of protracted bronchitis.

• Reversible airway obstruction: In accordance with American Thoracic Society and European Respiratory Society criteria with Australian predicted values used [23].

• Secondary diagnosis: Diagnosis found on objective tests but which: 1) specific treatment did not result in resolution or improvement of the cough; or 2) no treatment for this diagnosis was trialled and the cough resolved with other treatment or spontaneously [22].

• Specific cough pointers: Presence of any of the following- auscultatory abnormality, classical cough characteristics, cardiac abnormalities, chest pain, chest wall deformity, daily moist or productive cough for >3 months, digital clubbing, dyspnoea (exertional or at rest), failure to thrive, feeding difficulties (including choking/vomiting), haemoptysis, immune deficiency, neurodevelopmental abnormality, recurrent pneumonia, wheeze. These pointers are explained in TSANZ position statement [20].

• Spirometry abnormality: As determined by the American Thoracic Society and European Respiratory Society criteria with Australian predicted values used [23].

• Tertiary hospital management: Management that usually requires the level of investigations at a paediatric tertiary centre (bronchoscopy, chest HRCT scan, fluoroscopic swallowing screening, etc).

### Description of instruments for outcome measures

• Parent(s) proxy cough-specific QOL (PC-QOL): A validated multi-dimensional QOL designed for parents. This was modelled on Juniper's paediatric asthma QOL [24]. Each of the questions has a 7 point score, from one (worst) and 7 (best QOL). The scores to all the questions are added and the average is taken, thus one represents worst QOL and 7 represents best QOL [25,26].

• Cough diary: This validated scale is a verbal category descriptive score ranging from 0 (no cough) to 5 (cannot perform most usual activities due to severe coughing) [27].

• PedsQL™ 4.0[28]: This parent-proxy 23 item report for children is a generic validated multi-dimensional QOL designed for parental reports of their child's QOL. It is divided into 4 age groups (2-4, 5-7, 8-12, 13-18 years).

### Outcome measures

Primary outcomes for RCT: (a) proportion of children who are cough free; and (b) difference in PC-QOL at week 6 (with the intervention group being managed according to the pathway for at least 4 weeks). Secondary outcomes: change in PedsQL scores; proportion where a primary diagnosis was achieved; agreement of implementation of each step of the pathway (in the subset of children whose medical records were re-examined); clinical failure rate; diagnosis reached in number of children within the time frame of 12 months of enrolment; and proportion with recurrence of chronic cough related to original primary diagnosis (ie. possible misdiagnosis). As there are no published data on the outcomes of chronic cough in Indigenous children, the data from Indigenous children will be described separately.

### Sample size

To optimise the generalisability of the clinical management pathway, all children presenting to the clinicians over a 2 1/2 year period will be invited to participate. We anticipate enrolling approximately 220-250 children. This provides a sufficient power to address both primary outcomes. For a sample size of 220, the power to detect an improvement rate in the proportion of children whose cough has resolved[29] at week 6 from 35% to 55% using the pathway (compared to non-use of pathway) is 82% (significance 5%). For PC-QOL difference between groups of 1 (a clinically significant difference) assuming a SD of 1.05, a sample size of 50 (25 per group) yields a study power of 91%.

### Statistical analysis and reporting

Data will be reported and presented in accordance with the updated CONSORT criteria [30]. Children will be analysed according to allocation status (regardless of subsequent management). Clinical improvement will be defined by comparing: (a) the proportions of children who are cough free; and (b) the difference in PC-QOL at week 6 (with the intervention group being managed according to the pathway for at least 4 weeks). Efficacy of the clinical management pathway will be determined by improvements in both QOL measures and the percentage of children with a primary diagnosis achieved. Reliability will be determined by agreement in the implementation at each step of the pathway in children who have their medical records were re-examined. A kappa value of >0.6 will be considered acceptable for clinical practice (as recommended) [31]. Validity of the clinical pathway will be assessed by clinical failure rate (defined above), diagnosis reached by 12 months, and rates of misdiagnosis.

Unpaired and paired Student's T test will be used for continuous data comparisons (assuming normal distribution) and ANOVA utilised when >2 groups are compared. If data are not normally distributed, non parametric tests will be substituted. Chi squared tests will be used for categorical data. The 95% confidence intervals of all differences between the 2 study groups will be described.

### Ethical approval

The protocol has been granted full ethical approval from the respective Human Research Ethics Committees of all the participating institutions [Royal Children's Hospital (Brisbane), Royal Children's Hospital (Melbourne), The Children's Hospital at Westmead (Sydney), Canberra Hospital (Canberra), Royal Darwin Hospital (Darwin), and the Menzies School of Health Research (Darwin)].

## Discussion

Chronic cough is important because it causes a significant burden of distress to parents and may reflect a serious underlying serious disorder. Determining which children require further investigations and/or treatment is a key management issue. High quality research is required to determine who will benefit from which intervention and management approaches (including 'watchful waiting'). If use of the clinical pathway shows a clinically important benefit, this study will represent a substantial advance in the evidence on the management of chronic cough in children. Further, the results will influence clinical policy and form the basis of an updated clinical practice guideline. A standardised clinical management pathway has the potential to reduce the morbidity of chronic cough, unnecessary costs, and adverse events associated with medication use. As chronic lung disease often presents with cough, this pathway may also ensure earlier detection of serious respiratory problems.

### The rationale for the clinical pathway being studied and study outcome measures

Our clinical management pathway is based on:

• Systematic evaluation of the evidence with low susceptibility to bias updated from previous publications (in particular relevant Cochrane Reviews)[22]

• Definitions and key management approaches used in the Thoracic Society of Australia and New Zealand Position Statement [20].

• The findings of previous research on cough in children where we have shown that dry cough is more likely to resolve naturally [19].

• Where no evidence exists, management is based on clinical experience (approximately 250 doctor-years in total).

Objective cough monitoring is the gold standard for cough studies [27]. However in a clinical multi-centre study, cough monitors are not feasible. It is also arguable that QOL measures are more clinically relevant [32]. Thus we have chosen a parent cough-specific QOL (PC-QOL) [25]. Since the presence of cough influences QOL [33], the time taken for cough resolution is also a clinically important outcome.

### Limitations of our study

In addition to the lack of an objective measure of cough as an outcome measure, our study could not be performed as a double blinded study. Ideally, the RCT should involve the use vs. non-use of the clinical pathway and assess both short-term and long-term outcomes. However, our studies over the last decade have already influenced practice. Most specialists would not feel comfortable withholding investigation and treatment for a prolonged period. We have therefore designed a RCT to assess short-term outcomes only (at 6 weeks after initial referral). This is acceptable given the usual waiting time to see a specialist. Importantly, our design also allows us to assess short-term harm that might be associated with early referral (such as over-investigation and treatment of a spontaneously resolving illness).

In summary, this will be the first multicentre study (conducted in 5 major Australian cities) to examine the effectiveness of an evidence-based clinical pathway for the management of chronic cough in children.

## List of abbreviations

PC-QOL: Parent-proxy cough-specific QOL; PedsQL™ 4.0: Pediatric QOL; QOL: Quality of life; RCT: Randomised controlled trial; TSANZ: Thoracic Society of Australia and New Zealand.

## Competing interests

The author(s) declare that they have no financial competing interests. AC, CM, LL, PvA, NG and CR declare that the cough pathways were designed based on a cough guideline they co-authored and hence potentially have an intellectual competing interest.

## Authors' contributions

AC conceived the study, and participated in its design and coordination and drafted the manuscript. PM participated in its design and statistical analysis plan. PvA, CM, LL, CR, NG, IM and IT participated in its design. All authors read and approved the final manuscript.

## Funding

Study is funded by a 3-year Australian National Health and Medical Research Council (NHMRC) project grant (490321). Prof Chang is supported by a NHMRC practitioner fellowship (545216).
